# Supplementary material for: Reverse Phase Protein Array Profiling Identifies Recurrent Protein Expression Patterns of DNA Damage-Related Proteins across Acute and Chronic Leukemia: Samples from Adults and the Children’s Oncology Group
Source: Int J Mol Sci. 2023 Mar 13;24(6):5460. doi: 10.3390/ijms24065460 (PMC10056740; doi:10.3390/ijms24065460)
Supplement: Supplementary file 1 [file ijms-24-05460-s001.zip › ijms-2226086-supplementary.pdf]

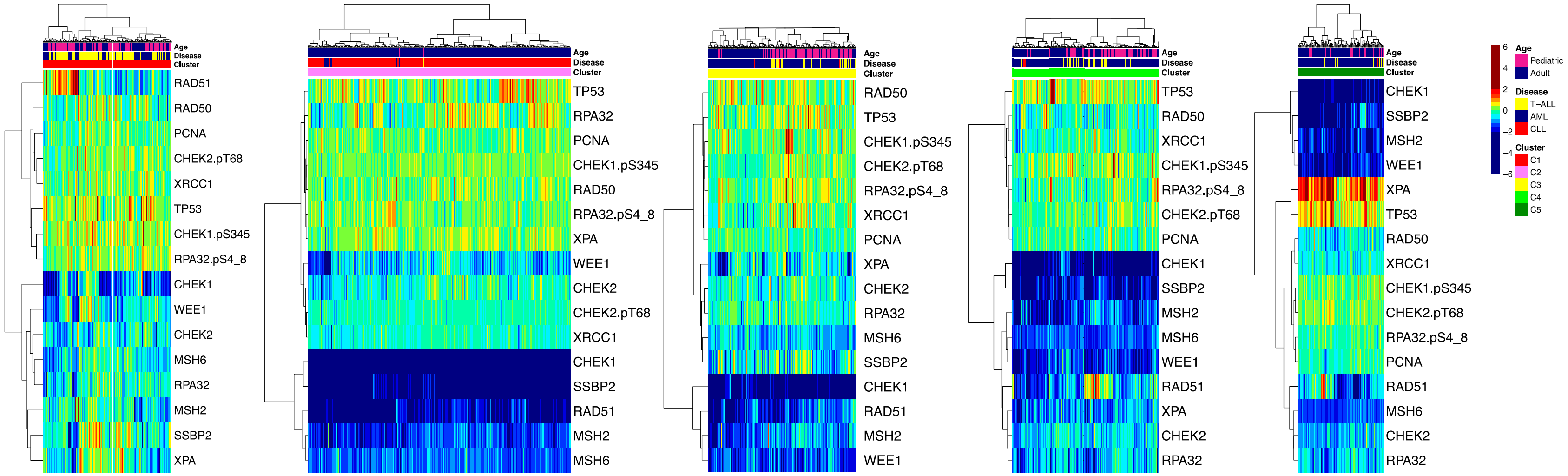

Supplemental Figure S1. Unbiased hierarchical clustering within each individual cluster. Unbiased hierarchical clustering was performed within each cluster to determine if subclusters based on age or disease would emerge.

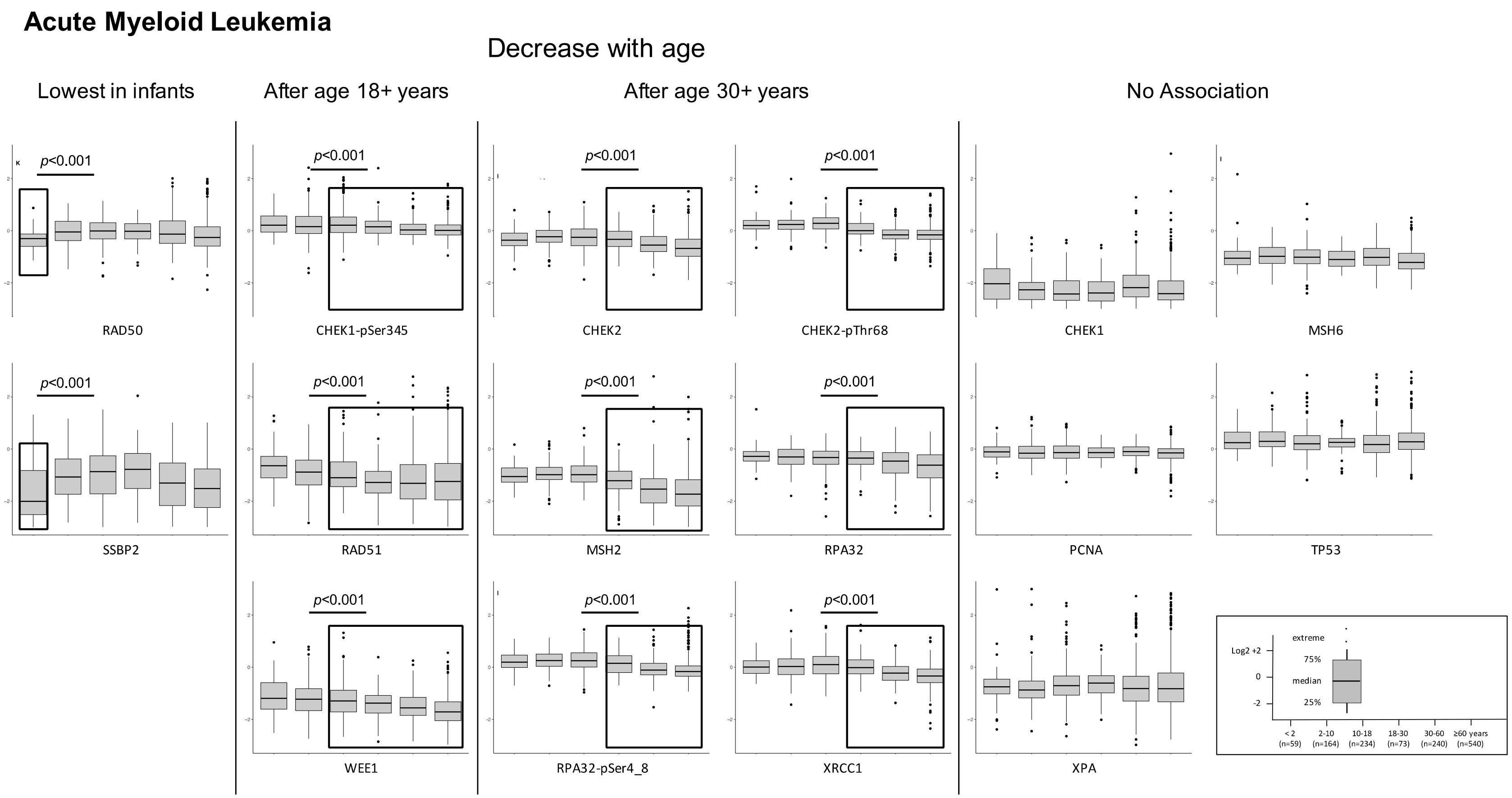

Supplemental Figure S2. DDR protein expression by age in AML. The expression of the individual proteins divided into six age categories: < 2 year of age, 2-10 years or age, 10-18 years or age, 18-30 years of age, 30-60 years or age and > 60 years of age.

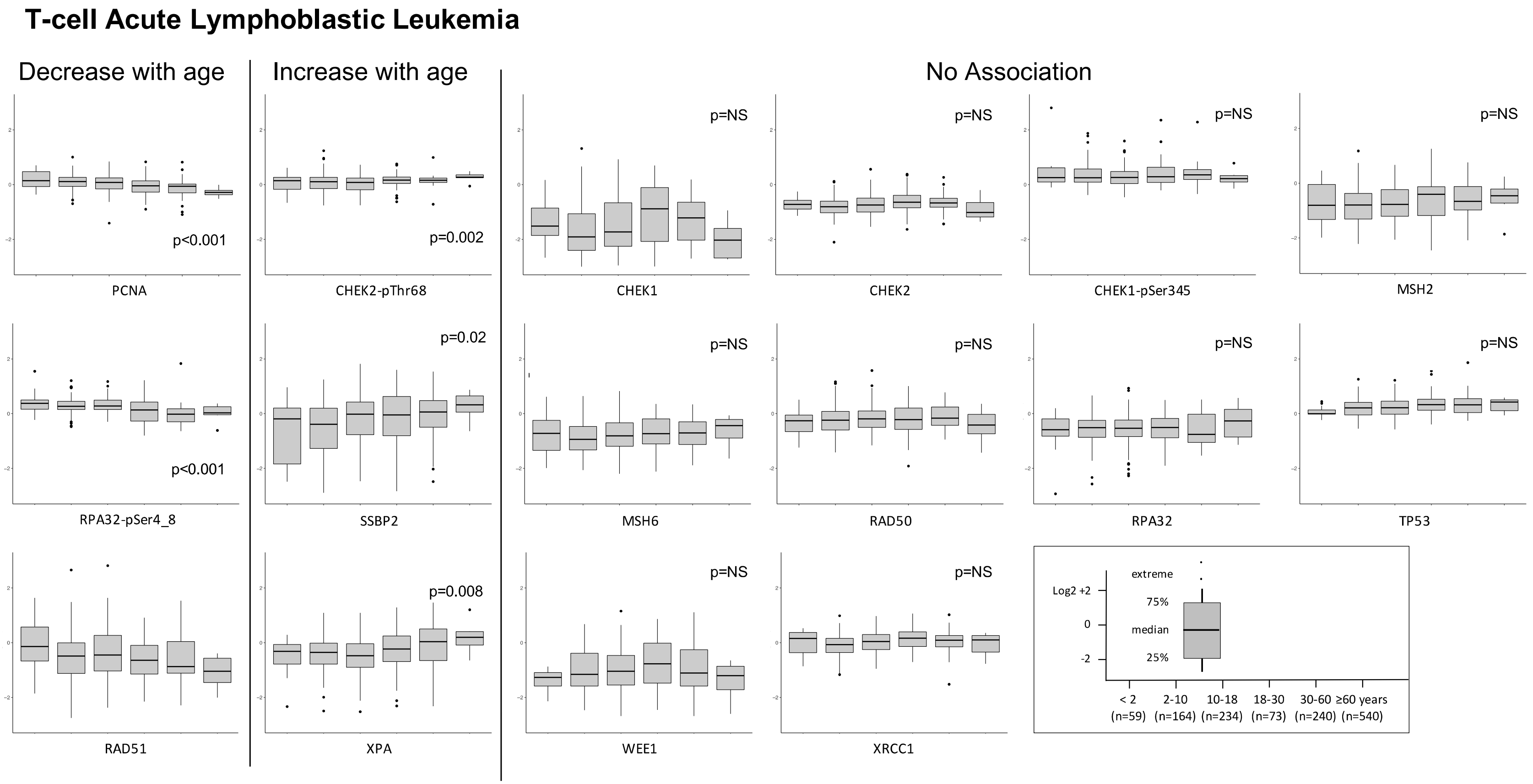

Supplemental Figure S3. DDR protein expression by age in T-cell ALL. The expression of the individual proteins divided into six age categories: < 2 year of age, 2-10 years of age, 10-18 years of age, 18-30 years of age, 30-60 years of age and > 60 years of age.

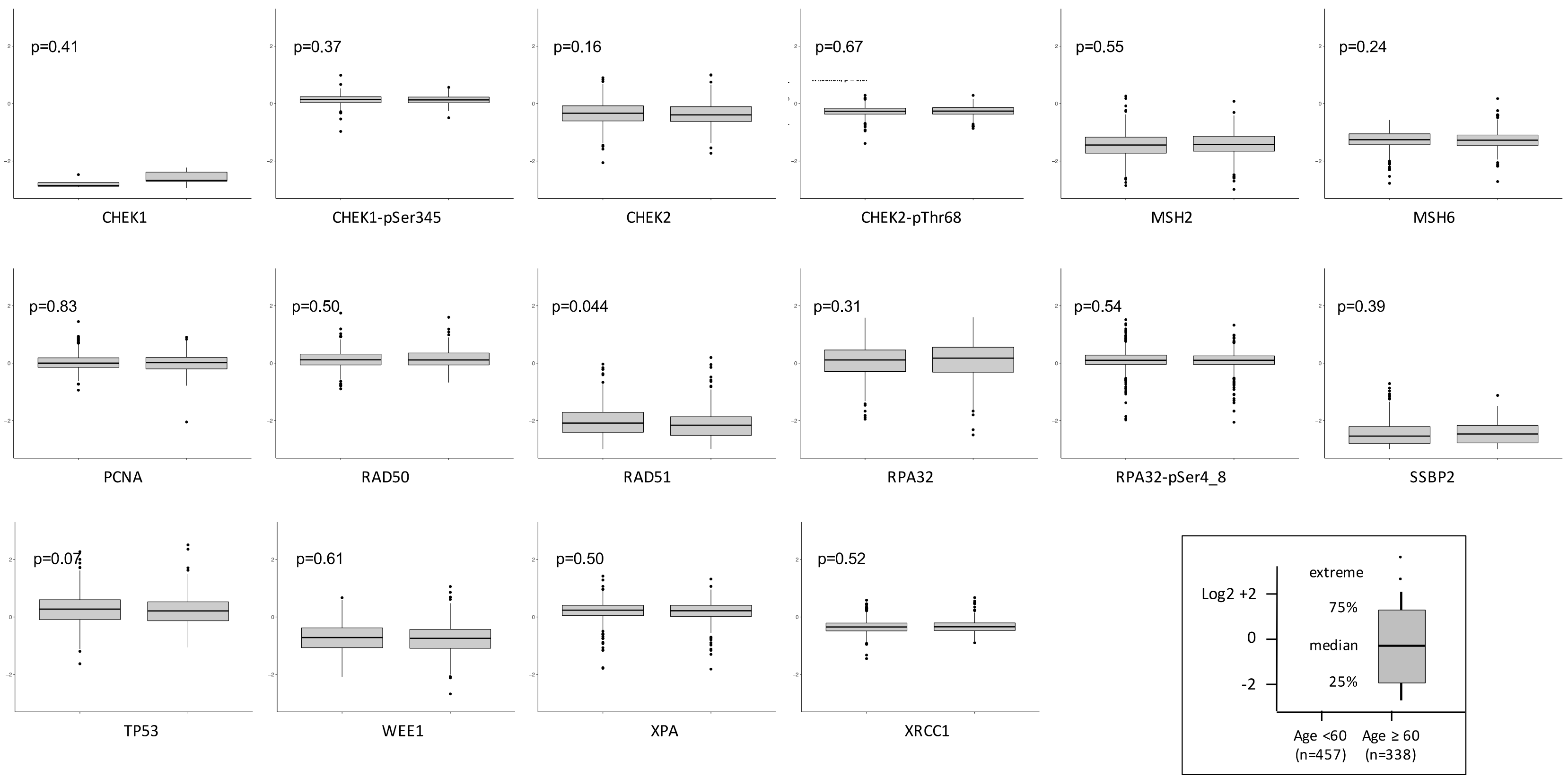

Supplemental Figure S4. DDR protein expression by age in CLL. The expression of the individual proteins divided into two age categories: < 60 year of age and ≥ 60 years of age.

Supplemental Table S1

AML	
AAML1031: ADE	243
AAML1031: ADE + bortezomib	212
AAML1031: ADE + sorafenib	36
ADE-based	9
2006-0813 IDA+HDAC	1
2007-0147 FLAG+Ida	26
2007-0147 FLAG+Ida+Mylo	1
2007-0147 FLAG+MYLO	5
2007-0685 Azacitidine	3
2007-0685 SAHA+Aza	4
2007-0727 SAPACITABINE	1
2007-0835 IA+SAHA	4
2009-0467 Azacitidine+Lenalidomide	1
2009-0536 Plerixafor+Clofarabine	4
2010-0374 Azacitidine+PKC412	4
2010-0511 Azacitidine+Sorafenib	2
2010-0615 SGI-110	13
2010-0727 DAC 5 alt Sapa	1
2010-0727 Decitabine 5	4
2010-0727 Sapacitabine	1
2010-0736 OM+LD AC	6
2010-0788 CIA	49
2010-0788 FAI	28
2010-0981 Arsenic+ATRA+Mylo	2
2011-0188 Tosedostat+Ara-C	1

CLL	
Untreated	476
ACP-196	6
AMD3100+Rituximab	1
AVL-292	1
BGB-3111	1
BR	1
CAL-101+Rituximab	6
CC-122+Obinutuzumab	1
CFAR	1
Chlorambucil	1
Cirmtuzumab+Ibrutinib	1
DASATINIB	1
Decitabine	2
EPOCH-R+Venetoclax	1
FCM-R???	1
FCM-R+Neulasta	1
FCR	118
FCR+GM-CSF	1
FCR3	3
Ibrutinib	17
Ibrutinib+Nivolumab	2
Ibrutinib+Rituximab	3
Ibrutinib+Venetoclax	5
IdKLH+GM-CSF	2
iFCG	18

TALL	
AALL1231: AFBM	139
AALL1231: AFBM + bortezomib	150
AAL1231: unknown	3
HCVAD+Nelarabine	28
HCVAD	23
Augmented BFM	10
Augemented HCVAD	3
CLIA1+Sorafenib	1
Clofa+Etop+Ctx+Vcr+Bortez	1
MiniHCVD+Venetoclax	1
Nelarabine	1
VAD	1

2011-0660 Clofa+Ara-C(LD)	5
2011-0987 Cladribine+Ara-C(LD)	35
2012-0062 PF-04449913+LD Ara-C	1
2012-0262 DFP-10917	2
2012-0569 Crenolanib	1
2012-0648 CLIA+Veneto+Midostaurin	1
2012-0648 CLIA+Venetoclax	12
2012-0648 CLIA1	17
2012-0648 CLIA1+Sorafenib	13
2012-0648 CLIA2	10
2012-0648 CLIA2+Midostaurin	1
2012-0648 CLIA2+Sorafenib	1
2012-0980 CPX-351	4
2012-0980 Dnr+Ara-C(LD)	2
2012-1017 Decitabine 10	18
2012-1017 Decitabine 5	11
2012-1047 Quizartinib+Aza	7
2012-1047 Quizartinib+LD Ara-c	4
2012-1064 DAC/CIA	1
2013-0099 Decitabine+Vosaroxin	22
2013-0416 Ara-C(LD)+Volasertib	1
2013-0596 Azacitidine+Pracinostat	1
2013-0843 SGI-110 10	3
2013-0843 SGI-110 5	6
2013-0843 SGI-110 5+Cladribine	3
2013-0843 SGI-110 5+Idarubicin	5
2013-0870 Omacetaxine	1
2013-0873 Azacitidine+Pracinostat	1

INVAC-1	4
IPI-145	2
JCAR017	2
KINETIC BIOMARKER	1
Lenalidomide	4
Lenalidomide+Ofatumumab	2
Lenalidomide+Rituximab	15
Obinutuzumab+GDC-0199	1
Obinutuzumab>Atezolizumab	2
Ofatumumab	13
PCI-32765	15
PCI-32765+BR	1
PCI-32765+Rituximab	3
Revlimid+Rituximab	1
Ritux+GM-CSF	5
Rituximab	5
Rituximab+Lirilumab	1
Rituximab+Steroids	4
Ruxolitinib	6
SCR	1
Venetoclax+CYC065	1
Venetoclax+Ibrutinib	35

2013-0901 SGI-110	1
2014-0076 Azacitidine+Sorafenib	5
2014-0344 Ruxolitinib+Decitabine	5
2014-0391 BVD-523	1
2014-0490 ABT-199+Azacitidine	3
2014-0490 ABT-199+Decitabine	8
2014-0548 CPX-351	16
2014-0615 Azacitidine+SGN-CD33A	1
2014-0615 Decitabine(5)+SGN-CD33A	2
2014-0615 SGN-CD33A	2
2014-0800 AG-120	4
2014-0861 Aza+Nivo+Ipilimumab	2
2014-0861 Azacitidine+Nivolumab	12
2014-0907 IA+Nivolumab	10
2014-1051 SGI-110 5	2
2015-0360 Clofa+Ara-C(LD)	1
2015-0360 Rigosertib	1
2015-0516 FT-1101	4
2015-0767 3(D)+7+AG-221	1
2015-0767 3(I)+7+AG-120	1
2015-0767 3(I)+7+AG-221	3
2015-1056 Azacitidine+AG-221	1
2016-0102 Decitabine(5)+SGN-CD33A	2
2016-0128 BP1001+Decitabine	2
2016-0128 BP1001+LD Ara-C	1
2016-0150 Azacitidine+FT-2102	1
2016-0198 Decitabine+Inecalcitol	1
2016-0646 AMV564	1

2016-0785	1
2016-0944 Azacitidine+Venetoclax	1
2016-0979 FLAG+Ida+Venetoclax	9
2016-0985 Azacitidine+Venetoclax	1
2017-0092 Ulocuplumab+Ara-C(LD)	3
2017-0173 Azacitidine+Pracinostat	1
2017-0337 Mylotarg+Glasdegib	1
2017-0337-Arm C	2
2017-0337-Arm D	2
2017-0398 Venetoclax+Ara-C(LD)	2
2017-0490 Aza+AG-120+Venetoclax	4
2017-0795 Hu5F9-G4+Azacitidine	3
2017-0912 Dec+Veneto+Gilteritinib	2
2017-0912 Dec+Veneto+Soraf	1
2017-0912 Decitabine+Venetoclax	28
2017-0937 CLIA+Dexra+Gilter+Venetoclax	1
2017-0937 CLIA+Mylo+Dexrazoxane	1
2017-0938	1
2018-0020 Clad+Ara-C(LD)+Venetoclax	17
2018-0235 CPX-351+Mylotarg	3
2018-0394 Quizartinib+Decitabine+Venetoclax	1
2018-0499 Aza+Enasidenib+Gilteritinib	1
2018-0499 Aza+Enasidenib+Venetoclax	1
2018-0499 Azacitidine+Enasidenib	1
2018-0724 Aza+Venetoclax+Pevonedistat	9
2019-0241 CYC065+Venetoclax	1
APR-246+Aza+Venetoclax	2
CLIA+Veneto+Gilteritinib	1

Off - Aza	1
Off - Aza Azacitidine+Sorafenib	5
OFF - BID FA	1
Off - CLIA CLIA	11
Off - CLIA CLIA+Midostaurin	1
Off - CLIA CLIA+Sorafenib	1
Off - CLIA CLIA+Venetoclax	6
Off - CLIA CLIA2	2
Off - CPX-351 CPX-351+Mylo+Venetoclax	4
Off - CPX-351 CPX-351+Venetoclax	1
Off - Dac	1
Off - Dac Dac+Mylo+Veneto	1
Off - Dac Dac+Ruxo+Veneto	1
Off - Dac Dac+Veneto+Gilteritinib	1
Off - Dac Decitabine	20
Off - Dac Decitabine+Ruxolitinib	2
Off - Dac Decitabine+Sorafenib	2
Off - Dac Decitabine+Venetoclax	9
Off - FAI FAI	4
Off - FAI FAI+Sorafenib	1
Off - FAI FAI+Venetoclax	1
Off - FLAG FLAG+Ida	8
Off - FLAG FLAG+Ida+Mylo	1
Off - FLAG FLAG+Ida+Venetoclax	1
Off - FLAG FLAG+Mylotarg	1
Off - FLAG FLAG+Venetoclax	1
Off - IA IA	8
Off - IA IA+Sorafenib	1

Off - MEC MEC	1
Off - Ruxo Ruxolitinib+Decitabine	1
Off - Venetoclax Ara-C(LD)+Mylo+Veneto	1
Off - Venetoclax Aza+Venetoclax	3
Off - Venetoclax Venetoclax+Ara-C(LD)	1
Off Ara-C(HD)	1
Off Ara-C(LD)	1
Off ARSENIC+ATRA	1
Off BID FA	3
Off BID FA+Sorafenib	1
Off CIA	4
Off Clad+Ara-C(LD)+Venetoclax	3
Off Clad+LD AC>Dac	3
Off Cladribine+Ara-C(LD)	3
Off CLOFA+LDAC	3
Off DAC	2
Off Decitabine+Sorafenib	1
Off Decitabine+Venetoclax	1
Off FAI	2
Off FLAG+IDA	2
Off IA	5
Off Ida+Ara-C(LD)+Imatinib	1
Off IDA+HDAC	1
SWOG1203 3+7	1
SWOG1203 IA+V	1
Unknown	121

Supplemental Table S2.

Protein Name Rosetta Stone					RPPA Staining Details											
**Antibody name**	**RPPA Antibody name**	**HUGO name (added with PTM)**	**MiMI name**	**Full description/ GeneCards name**	**R2, WB vs. RPPA**	**Host**	**Company**	**Catalog**	**Primary dilution**	**Secondary dilution**	**Primary dilution**	**Secondary dilution**	**Primary dilution**	**Secondary dilution**	**Primary dilution**	**Secondary dilution**
									**Pedi-AML**		**T-ALL**		**CLL**		**Adult AML**	
ATM	ATM	ATM	ATM	ATM Serine/Threonine Kinase	> 0.7	Rabbit	Cell Signaling	2873	750	20000	750	18000	750	10000		
Phospho-ATM (Ser1981)	ATM.pS 1981	ATM Phospho Ser 1981	ATM	ATM Serine/Threonine Kinase	> 0.7	Rabbit	Cell Signaling	5883	250	20000	100	15000	150	10000		
Aurora A	AURKA	AURKA	AURKA	Aurora Kinase A	> 0.7	Rabbit	Cell Signaling	3094					400	10000		
MERIT40 (Phospho S29)	BABAM1 -pSer29	BABAM1 phospho Serine 29	BABAM1	BRISC And BRCA1 A Complex Member 1	> 0.7	Rabbit	Cell Signaling	9154	50							
Anti-Bap1	BAP1	BAP1	BAP1	BRCA1 Associated Protein 1	> 0.7	Mouse	Santa Cruz	sc-28383					250	10000		
BRCA2	BRCA2	BRCA2	BRCA2	BRCA2, DNA Repair Associated	0.5 - 0.7	Rabbit	Cell Signaling	9012	100	20000	75	15000	75	10000		
Cdc25c	CDC25C	CDC25C	CDC25C	Cell Division Cycle 25C	>0.7	Rabbit	Cell Signaling	4688			200	18000	200	10000	75	10000
CDK9	CDK9	CDK9	CDK9	Cyclin Dependent Kinase 9	>0.7	Rabbit	Cell Signaling	2316							2000	10000
CDT1	CDT1	CDT1	CDT1	Chromatin Licensing And DNA Replication Factor 1	>0.7	Rabbit	Cell Signaling	8064							100	10000
Chk1	CHEK1	CHEK1	CHEK1	Checkpoint Kinase 1	0.5 - 0.7	Mouse	Cell Signaling	2360	500	15000	400	18000	500	10000	250	10000
Phospho-Chk1 (Ser345)	CHEK1-perS345	CHEK1 Phospho Ser 345	CHEK1	Checkpoint Kinase 1	0.5 - 0.7	Rabbit	Cell Signaling	2348	100	20000	200	18000	250	10000	250	10000
Chk2	CHEK2	CHEK2	CHEK2	Checkpoint Kinase 2	> 0.7	Mouse	Cell Signaling	3440	150	20000	150	18000	150	10000	150	10000

Phospho-Chk2 (Thr68)	CHEK2-pThr68	CHEK2 Phospho Thr 68	CHEK2	Checkpoint Kinase 2	0.5 - 0.7	Rabbit	Cell Signaling	2197	500	20000			500	10000	500	10000
DDB-1	DDB1	DDB1	DDB1	Damage Specific DNA Binding Protein 1	>0.7	Rabbit	Cell Signaling	6998					800	10000	400	10000
ERCC1	ERCC1	ERCC1	ERCC1	ERCC Excision Repair 1, Endonuclease Non-Catalytic Subunit	> 0.7	Mouse	Santa Cruz Biotechnol<br>ogy	sc-<br>17809	75	18000	75	18000	75	10000		
ERCC5	ERCC5	ERCC5	ERCC5	ERCC Excision Repair 5, Endonuclease Non-Catalytic Subunit	0.5-<br>0.7	Rabbit	Protein Tech	11331-<br>1-AP	300	18000	750	18000	750	10000		
Phospho-Histone H2A.X (Ser140)	H2AX.pS<br>140	H2AX Phospho Ser 140	H2AX	H2A Histone Family Member X	0.5 - 0.7	Mouse	Thermo Fisher Scientific	MA1-<br>2022	400	18000			1000	10000		
MSH2	MSH2	MSH2	MSH2	MutS Homolog 2	> 0.7	Mouse	Cell Signaling	2850	150	25000	75	18000	150	10000	150	10000
MSH6	MSH6	MSH6	MSH6	MutS Homolog 6	0.5 - 0.7	Rabbit	Novus Biologicals	220300<br>02	2000	20000	800	18000	2000	10000	2000	10000
PARP	PARP1	PARP1	PARP1	Poly(ADP-Ribose) Polymerase 1	0.5 - 0.7	Rabbit	Cell Signaling	9542	200	18000	200	18000	200	10000	400	10000
Anti-PCNA antibody	PCNA	PCNA	PCNA	Proliferating Cell Nuclear Antigen	0.5 - 0.7	Rabbit	Abcam	ab9255<br>2	500	18000			500	10000	500	10000
PD-1	PDCD1	PDCD1	PDCD1	Programmed Cell Death 1	>0.7	mouse	Cell Signaling	43248			50	15000	50	10000	50	10000
Anti-Rad50	RAD50	RAD50	RAD50	RAD50 Double Strand Break Repair Protein	> 0.7	Mouse	Merck	05-525	2000	20000	2000	18000	2000	10000	2000	10000
Rad51	RAD51	RAD51	RAD51	RAD51 Recombinase	> 0.7	Rabbit	Cell Signaling	8875	200	18000	75	18000	100	10000	100	10000
RPA32/RPA2	RPA32	RPA32	RPA32	Replication Protein A2	0.5 - 0.7	Rat	Cell Signaling	2208	2000	30000	2000	30000	2000	10000	2000	10000
Phospho RPA32 (S4/S8)	RPA32-pSer4_8	RPA32 Phospho Ser 4/8	RPA32	Replication Protein A2	0.5 - 0.7	Rabbit	Bethyl	A300-<br>245A	2000	25000	2000	25000	2000	10000	2000	10000
Anti-SIRT1	SIRT1	SIRT1	SIRT1	Sirtuin 1	0.5 - 0.7	Rabbit	Abcam	ab3244<br>1	1000	15000	2000	18000	2000	10000	800	10000
SirT6	SIRT6	SIRT6	SIRT6	Sirtuin 6	> 0.7	Rabbit	Cell Signaling	2590	800	20000	500	18000	500	10000	500	10000
Anti-SSBP2	SSBP2	SSBP2	SSBP2	Single Stranded DNA Binding Protein 2	0.5 - 0.7	Rabbit	Abcam	ab1779<br>44	1000	20000	1000	18000	1000	10000	500	10000
p53	TP53	TP53	TP53	Tumor Protein P53	0.5 - 0.7	Rabbit	Cell Signaling	9282	1000	20000	500	18000	2000	10000	2000	10000
53BP1	TP53BP1	TP53BP1	TP53BP1	Tumor Protein P53 Binding Protein 1	> 0.7	Rabbit	Cell Signaling	4937					300	10000	300	10000

Wee1	WEE1	WEE1	WEE1	WEE1 G2 Checkpoint Kinase	0.5 - 0.7	Rabbit	Cell Signaling	4936	500	20000	500	18000	500	10000	300	10000
XPA	XPA	XPA	XPA	XPA, DNA Damage Recognition And Repair Factor	0.5 - 0.7	Mouse	Santa Cruz Biotechnology	sc-56813	75	20000	75	18000	75	10000	150	10000
XPF	XPF	XPF	XPF	XPF, DNA Damage Recognition And Repair Factor	0.5 - 0.7	Rabbit	Abcam	ab73720			50	15000	50	10000	150	10000
Ku80	XRCC1	XRCC1	XRCC1	X-Ray Repair Cross Complementing 1	0.5 - 0.7	Rabbit	Cell Signaling	2753	150	18000	100	18000	200	10000	200	10000
